# Proteolytic Activation of the Epithelial Sodium Channel (ENaC): Its Mechanisms and Implications

**DOI:** 10.3390/ijms242417563

**Published:** 2023-12-16

**Authors:** Mohammed Aufy, Ahmed M. Hussein, Tamara Stojanovic, Christian R. Studenik, Mohamed H. Kotob

**Affiliations:** 1 Division of Pharmacology and Toxicology, Department of Pharmaceutical Sciences, University of Vienna, 1090 Vienna, Austria; ahmed.hussein@univie.ac.at (A.M.H.); kotobm84@univie.ac.at (M.H.K.); 2 Department of Zoology, Faculty of Science, Al-Azhar University, Assiut 71524, Egypt; 3 Programme for Proteomics, Paracelsus Medical University, 5020 Salzburg, Austria; tamara.stojanovich@gmail.com; 4 Department of Pathology, Faculty of Veterinary Medicine, Assiut University, Assiut 71515, Egypt

**Keywords:** ENaC, ENaC activation, proteases, epithelial sodium channel, furin, plasmin, prostasin, kallikrein, cathepsin S, cathepsin B

## Abstract

Epithelial sodium channel (ENaC) are integral to maintaining salt and water homeostasis in various biological tissues, including the kidney, lung, and colon. They enable the selective reabsorption of sodium ions, which is a process critical for controlling blood pressure, electrolyte balance, and overall fluid volume. ENaC activity is finely controlled through proteolytic activation, a process wherein specific enzymes, or proteases, cleave ENaC subunits, resulting in channel activation and increased sodium reabsorption. This regulatory mechanism plays a pivotal role in adapting sodium transport to different physiological conditions. In this review article, we provide an in-depth exploration of the role of proteolytic activation in regulating ENaC activity. We elucidate the involvement of various proteases, including furin-like convertases, cysteine, and serine proteases, and detail the precise cleavage sites and regulatory mechanisms underlying ENaC activation by these proteases. We also discuss the physiological implications of proteolytic ENaC activation, focusing on its involvement in blood pressure regulation, pulmonary function, and intestinal sodium absorption. Understanding the mechanisms and consequences of ENaC proteolytic activation provides valuable insights into the pathophysiology of various diseases, including hypertension, pulmonary disorders, and various gastrointestinal conditions. Moreover, we discuss the potential therapeutic avenues that emerge from understanding these mechanisms, offering new possibilities for managing diseases associated with ENaC dysfunction. In summary, this review provides a comprehensive discussion of the intricate interplay between proteases and ENaC, emphasizing the significance of proteolytic activation in maintaining sodium and fluid balance in both health and disease.

## 1. Introduction

**Figure 1:**
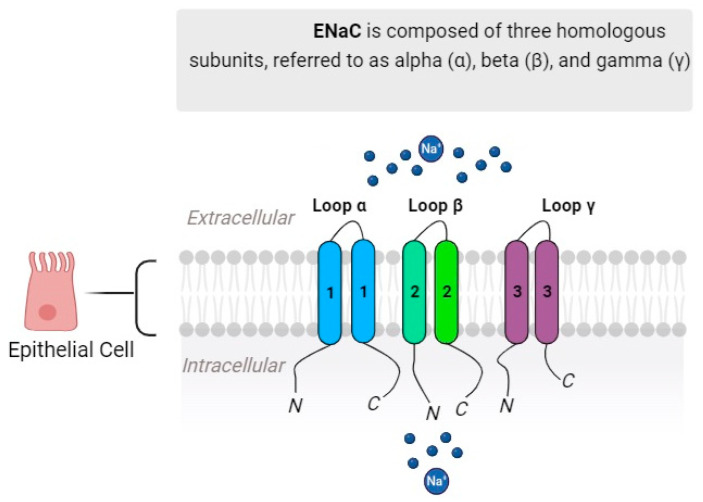
Canonical ENaC is composed of three homologous subunits referred to as alpha (α), beta (β), and gamma (γ). Together, these subunits combine to create a functional channel with multiple components, forming a heteromultimeric structure.

Epithelial sodium channel (ENaC) are integral membrane proteins that play a crucial role in the regulation of sodium reabsorption in various epithelial tissues [1,2]. ENaC consist of the following three homologous subunits: α, β, and γ (Figure 1), which form a functional channel complex responsible for the selective transport of sodium ions across the epithelial cell membranes [3,4]. In certain cell types, an additional fourth subunit known as δ-subunit is present. The prevailing concept is that the functional epithelial sodium channel (ENaC) must consist of at least one α or α-like subunit, with examples including δ and δ-like ε ENaC from *Xenopus laevis*. For optimal channel activity, both β and γ subunits are necessary, as they contribute to a significant increase in channel activity by up to two orders of magnitude [2]. In the exploration of ENaC, an integral component in ion channel physiology, a study was conducted that delves into the structural intricacies revealed by recent advances [5]. This study focused on the architecture of human ENaC in its uncleaved state, elucidated through single-particle cryo-electron microscopy. ENaC manifests as heterotrimeric channels, showcasing pivotal protease-sensitive domains that are crucial for effective channel gating. This structural analysis highlights a compelling assembly with a 1:1:1 stoichiometry of α:β:γ subunits arranged in a counter-clockwise orientation. Each subunit’s distinctive hand-like structure encompasses key gating domains, which are often referred to as a ‘finger’ and a ‘thumb.’ A notable revelation emerges with the identification of an elusive protease-sensitive inhibitory domain nestled between these crucial domains. This domain assumes a strategic role in orchestrating conformational changes within the ‘finger’ and ‘thumb’, offering an unprecedented view of the inhibitory architecture governing ENaC [5].

In contrast to ENaC subunits α and β, the δ subunit is more widely distributed and can be found in a variety of tissues, including both non-epithelial (such as the brain, heart, ganglion, placenta, blood, etc.) and epithelial tissues (including the trachea, pancreas, liver, stomach, etc.) [6,7,8,9,10,11,12,13,14,15,16,17,18,19]. ENaC are widely distributed in tissues such as the kidney, lung, colon, sweat glands, and salivary glands [2,20,21,22,23,24,25]. The primary function of ENaC is to reabsorb sodium from the luminal side of epithelial cells and transport it into the interstitial fluid [26]. This process is crucial for maintaining an electrolyte balance, blood pressure regulation, and overall control over fluid volume [27]. By controlling sodium reabsorption, ENaC play a key role in determining the concentration of sodium in the extracellular fluid and, consequently, the osmolarity and volume of certain organ fluids [28].

The activity of ENaC is tightly regulated to maintain sodium balance. However, the activation of ENaC can be triggered by certain peptides, such as the TIP peptide derived from the tumor necrosis factor (TNF). When this peptide binds to the α subunit of the ENaC channel, it initiates the activation process [29,30,31,32,33,34,35,36,37,38,39]. The activation of ENaC is further subject to multiple regulatory mechanisms, such as post-translational modifications, notably N-glycosylation, which plays a significant role in ENaC functioning [25]. When the glycosylation sites on the α-subunit of ENaC are ablated or removed, it disrupts the interaction between the TIP peptide and ENaC, resulting in the inhibition of α-ENaC activation by this particular peptide [25].

In addition, certain hormones play an important role in ENaC activation. Aldosterone, for instance, primarily acts in the distal parts of the kidney tubules to increase the abundance and activity of ENaC, which is responsible for mediating the entry of sodium ions (Na^+^) into the apical membrane of epithelial cells in these tubules [2]. This increased sodium reabsorption leads to greater sodium retention in the body, which, in turn, leads to increased water retention. This process ultimately helps to raise blood pressure and maintain fluid balance within the body [40,41,42,43,44].

Angiotensin is another example of the hormonal activation of ENaC. Angiotensin II (Ang II) is a hormone that plays a central role in the renin–angiotensin–aldosterone system (RAAS), a hormonal system that regulates blood pressure and fluid balance in the body. Ang II has several functions in the body, including the stimulation of various physiological processes in various organs that collectively increase the circulating volume and elevate systemic blood pressure [4]. In the kidney, it stimulates the renal tubular reabsorption of water and salt, which results in anti-natriuresis (decreased sodium excretion) and antidiuresis (decreased urine production) [45]. This helps the body retain sodium and water, which can contribute to an increase in blood pressure and the circulating volume. One herein important aspect of Ang II’s role in the kidney is its ability to enhance the activity of ENaC in the aldosterone-sensitive distal nephron, resulting in increased sodium reabsorption in the kidney tubules, further contributing to anti-natriuresis and antidiuresis [45]. It is worth noting that Ang II not only stimulates the secretion of aldosterone, a hormone that also enhances sodium reabsorption, but it can also directly increase ENaC activity, which has a more immediate impact on sodium transport in the kidney. In summary, Ang II is a key component of the RAAS and has a multifaceted role in regulating sodium and water balance in the kidney. Its effects on ENaC activation and other processes contribute to its ability to increase systemic blood pressure and maintain proper fluid balance in the body [46,47,48,49].

Another example of the hormonal regulation of ENaC is AVP, also referred to as vasopressin or the antidiuretic hormone, which primarily plays a key role in regulating water balance and blood pressure within the body. Its primary function is to promote water reabsorption in the kidney tubules, conserving water and concentrating urine. However, the activation of ENaC by AVP implies that it may also influence sodium reabsorption [50,51].

An additional important regulatory mechanism involved in ENaC activity is proteolytic activation by proteases [52]. The proteolytic processing of ENaC subunits by specific proteases leads to the activation of the channel and increased sodium transport [52,53]. This process plays a crucial role in fine-tuning ENaC activity and ensuring the appropriate reabsorption of sodium ions [52]. Channels that do not undergo proteolytic processing demonstrate a significantly diminished opening probability due to the inhibition of external sodium ions, which is a phenomenon known as *Na^+^ self-inhibition* [5].

The significance of proteolytic activation in regulating ENaC activity is underscored by the fact that the dysregulation of this process can lead to various pathological conditions [53,54]. Aberrant ENaC activation has been implicated in hypertension, cystic fibrosis, Liddle syndrome, and other disorders associated with sodium and fluid imbalance [55,56]. Therefore, understanding the mechanisms and physiological implications of the proteolytic activation of ENaCis of great importance for elucidating the pathophysiology of these diseases and exploring potential therapeutic targets.

In this review article, we aim to provide a comprehensive overview of the structure, function, and tissue distribution of ENaC. We emphasize the importance of ENaC in sodium reabsorption and the maintenance of electrolyte balance. Furthermore, we delve into the significance of proteolytic activation as a regulatory mechanism for ENaC activity. By examining the different proteases involved in ENaC activation and their specific cleavage sites, we shed light on the intricate interplay between proteolytic processing and the function of ENaC. Finally, we discuss the physiological implications of proteolytic ENaC activation, emphasizing its role in blood pressure regulation, pulmonary function, and intestinal sodium absorption.

The proteolytic enzymes are classified based on their active site residues into four classes, which are serine, cysteine, aspartic, and metalloproteinases [57,58,59]. Most players that participate in ENaC proteolytic activation are some members of serine and cysteine proteases [53,60,61,62]. Furin, prostasin, and tissue kallikrein are serine protease members that are implicated in ENaC activation [63,64,65,66]. While cathepsin B is the key player of cysteine proteases that participate in the proteolytic activation of ENaC [67,68]. In addition to cathepsin B, cathepsin S and calpain-I also play a vital role in ENaC activation [61,62].

Through this comprehensive analysis, we hope to enhance our understanding of the role of proteolytic activation in modulating ENaC activity and its implications for health and disease. Such insights may pave the way for the development of novel therapeutic strategies targeting proteolytic pathways to restore the ENaC function and alleviate the pathophysiology associated with sodium and fluid imbalance-related disorders.

## 2. Proteolytic Activation of ENaC

### 2.1. Furin-like Convertases

**Figure 2:**
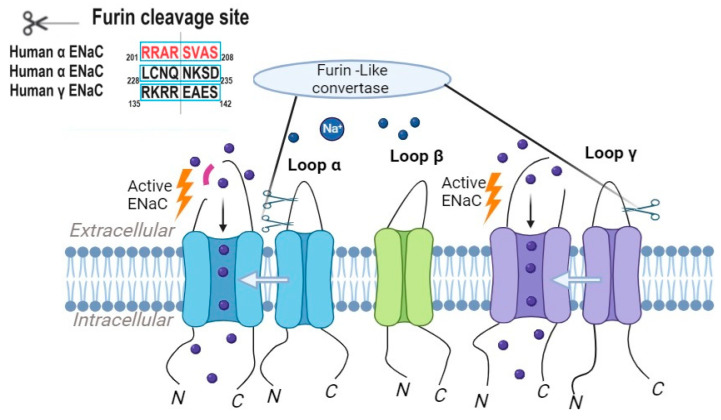
Proteolytic cleavage triggers the activation of ENaC, with furin initiating this process by cleaving the α subunit of ENaC twice and the γ subunit once.

Furin-like convertases are a family of proteases involved in the proteolytic activation of ENaC [69]. These convertases cleave the extracellular domains of ENaC subunits, resulting in channel activation [3]. The α and γ subunits of ENaC contain two and one furin cleavage consensus sites, respectively (Figure 2), and the mutation or deletion of any of these sites impairs channel activation [70]. Furin-like convertases, including furin itself and PC5/6, have emerged as pivotal regulators of ENaC activity in various tissues, including the kidney and lung [71]. The furin inhibitor BOS-318, through the selective inhibition of furin, effectively and markedly suppresses ENaC-mediated sodium transport in differentiated human bronchial epithelial cells. This inhibition was observed in both short-term (IC50 = 263.0 nM) and long-term (IC50 = 17.4 nM) treatment conditions [72], corroborating with previous investigations that indicate reduced basal ENaC activity following furin deficiency or inhibition [73,74]. The attenuation of ENaC activity has been correlated with favorable outcomes related to airway hydration and mucociliary clearance. It has been demonstrated that BOS-318-mediated ENaC inhibition leads to a simultaneous elevation in the height of the airway surface liquid and increased rates of mucociliary clearance in cystic fibrosis airway epithelial cells [72]. These collective findings suggest that furin targeting has the promising potential to offer therapeutic advantages in the management of cystic fibrosis.

### 2.2. Tissue Kallikreins

Other examples of key players in ENaC processing are tissue kallikreins, which are a family of serine proteases that are involved in the proteolytic activation of ENaC. Tissue kallikreins cleave the γ subunit of ENaC, leading to an increased channel open probability [75]. These proteases are expressed in various tissues, including the kidney and airway epithelia, and are implicated in the regulation of sodium reabsorption [76].

Tissue kallikrein knockout mice (TK^−/−^) are genetically modified mice where the TK gene is disrupted or deleted, resulting in reduced or absent TK activity and impaired γ-ENaC processing. When membrane proteins from the renal cortex of TK^−/−^ mice are incubated with TK in an in vitro setting, a 70 kDa band of gamma-ENaC appears [75]. This finding indicates that TK can promote γ-ENaC cleavage in vitro. In other words, the introduction of TK in an in vitro environment partially restores the processing of gamma-ENaC [75].

Among the tissue kallikreins, Kallikrein 1 (KLK1) has emerged as a key protease involved in the proteolytic activation of ENaC. KLK1 has been demonstrated to enhance the cleavage of γ-ENaC in the native collecting duct (CD), suggesting the potential direct involvement of this endogenous protease in the regulation of sodium (Na^+^) balance. This finding hints at the role of KLK1 in modulating γ-ENaC activity within the CD, which could impact sodium reabsorption and, subsequently, sodium homeostasis in the body [77]. This discovery may have implications for understanding and potentially managing the conditions related to sodium regulation, such as hypertension and electrolyte imbalances, although further research is necessary to confirm and fully comprehend these implications. In the lung, Kallikrein is predominantly produced by bronchial epithelial cells and has been implicated in the regulation of ENaC activity [75].

Regulation of KLK-Mediated ENaC Activation by Hormonal and Signaling Pathways

The activity of KLK7 in mediating ENaC proteolytic processing is regulated by hormonal and signaling pathways. One important regulator of KLK activity is the serine protease inhibitor LEKTI (lympho-epithelial Kazal-type-related inhibitor) [78]. LEKTI binds to and inhibits KLK, preventing its proteolytic activity on ENaC [78]. This regulatory interaction ensures precise control over ENaC activation via KLK.

Furthermore, several hormonal and signaling pathways have been shown to modulate KLK expression and activity, thereby influencing ENaC activation. For instance, the glucocorticoid hormone cortisol has been found to upregulate KLK expression in airway epithelial cells, leading to increased ENaC proteolytic processing and sodium transport [79]. This hormonal regulation of KLK provides a mechanism for the fine-tuning of ENaC activity in response to physiological and pathological conditions.

Overall, the proteolytic activation of ENaC by tissue kallikreins is subjected to regulation by various hormonal and signaling pathways. The presence of serine protease inhibitors, such as LEKTI, helps to fine-tune the activity of KLK and ensure precise control over ENaC activation. Hormones like cortisol have been shown to influence KLK expression and activity, thereby modulating ENaC proteolytic processing and sodium transport.

In conclusion, tissue kallikreins, particularly KLK, play a crucial role in the proteolytic activation of ENaC. Their ability to cleave the γ subunit of ENaC enhances the channel’s open probability and facilitates sodium reabsorption in various tissues. KLK activity is regulated by serine protease inhibitors and influenced by hormonal and signaling pathways. The dysregulation of KLK-mediated ENaC activation can contribute to the development of electrolyte imbalances and respiratory disorders. Further research is warranted to elucidate the intricate regulatory mechanisms involved in exploring the therapeutic potential of targeting KLK and related pathways for the treatment of ENaC-related disorders.

### 2.3. Prostasin

**Figure 3:**
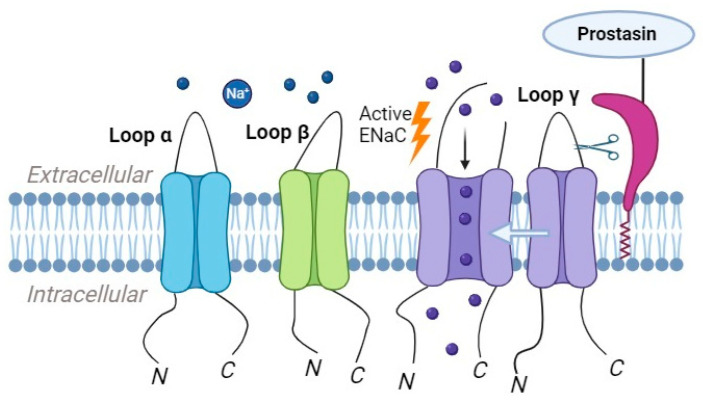
Prostasin has the capability to cleave ENaC, leading to the subsequent cleavage of the γ-subunit, which results in the release of an inhibitory peptide.

Prostasin, also known as channel-activating protease-1 (CAP1), a glycosylphosphatidylinositol-anchored serine protease, is present in the prostate gland, kidney, bronchi, colon, liver, lung, pancreas, and salivary glands. Its substrate specificity is trypsin-like [80]. It has been identified in nuclear and membrane fractions [81]. Prostasin’s activation of ENaC occurs through the cleavage of the γ-subunit, resulting in the release of an inhibitory peptide from the extracellular domain. The serine protease prostasin enhances ENaC activation by initiating the cleavage of the γ- subunit at a site located away from the furin cleavage site (Figure 3). Furin cleaves gamma ENaC at site 43, initiating a key molecular event. Subsequently, prostasin further refines this process by cleaving γ ENaC at site 186. ENaC channels that lack this specific furin and/or prostasin cleavage site exhibit increased activity, resulting in a higher open probability. Additionally, a synthetic peptide is designed to match the fragment cleaved from the gamma subunit functions as a reversible inhibitor of native ENaC in mouse cortical-collecting duct cells and primary cultures of human airway epithelial cells [80].

The activation of prostasin and its subsequent effect on ENaC activity are tightly regulated. Prostasin is initially synthesized as an inactive zymogen, and its activation involves the proteolytic cleavage of the propeptide domain. This cleavage can be mediated by other proteases, such as matriptase, which activates prostasin by cleaving to the propeptide domain [82].

Once activated, prostasin interacts with ENaC, leading to the proteolytic processing of the γ subunit RKRK186 site [63]. This processing enhances the channel’s open probability and promotes sodium transport. The exact mechanisms by which prostasin activates ENaC are not fully understood, but it is believed to involve changes in channel gating properties and interactions with other regulatory proteins [79].

The targeted deletion of prostasin in specific tissues, such as the lung and colon, in adult epithelial phenotypes has provided clear evidence implicating prostasin in ENaC-mediated sodium transport [83,84,85]. However, this association was not observed in the skin [85,86,87,88]. Notably, mice with an alveolar-specific prostasin knockout exhibited a 40% reduction in the ENaC-mediated Na^+^ current, leading to impaired alveolar fluid clearance. Nevertheless, unchallenged mice did not show alveolar edema, alterations in lung morphology, or changes in their tight junction protein abundance; these effects only manifested under increased hydrostatic pressure [83]. Similarly, a decrease in ENaC activity was noted in colon-specific prostasin knockout mice.

### 2.4. Plasmin as a Regulator of ENaC Activity

**Figure 4:**
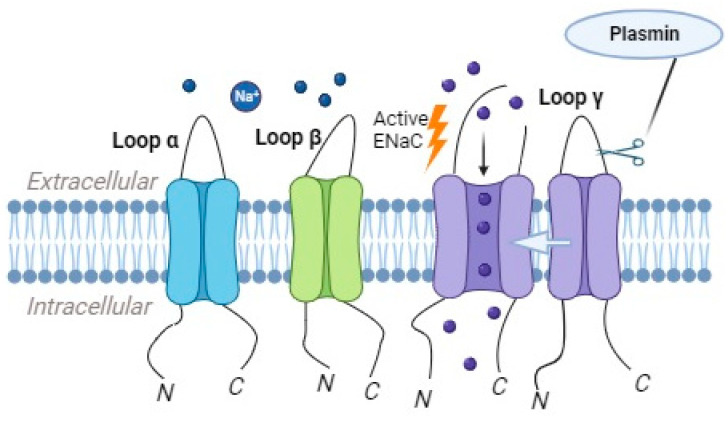
Plasmin has the ability to directly cleave the γ subunit of the epithelial sodium channel (ENaC), which results in the increased likelihood of the channel being open and consequently enhances the reabsorption of sodium ions.

Plasmin, as a highly potent and reactive serine protease, exhibits key roles in several physiological processes, such as thrombolysis, embryogenesis, cancer progression, and wound healing [89]. It is another serine protease that is implicated in the regulation of ENaC activity. This enzyme is derived from plasminogen through the action of plasminogen activators, such as the tissue-type plasminogen activator (tPA) or urokinase-type plasminogen activator (uPA) [82]. Plasmin can directly cleave the γ subunit of ENaC, leading to an increase in the channel’s open probability and enhanced sodium reabsorption (Figure 4) [90].

The activation of plasminogen and the subsequent generation of plasmin are tightly regulated by the presence of plasminogen activator inhibitors (PAIs) and other regulatory factors. The balance between plasminogen activators and their inhibitors determines the level of plasmin activity and its impact on the ENaC’s function.

In nephrotic syndrome, the involvement of plasmin in activating ENaC serves as a notable illustration. The suppression of plasma aldosterone in nephrotic syndrome has prompted investigations into the alternative pathway of epithelial sodium channel (ENaC) activation through extracellular proteolysis [91,92]. Urine samples from nephrotic syndrome patients have been found to contain soluble proteolytic activity, with plasmin identified as the major protease aberrantly filtrated from the plasma as a plasminogen [93,94]. In vitro studies have shown that exposure to nephrotic urine containing plasmin or purified plasmin enhances an inward amiloride-sensitive current in collecting duct cells [93,95]. Plasmin has been demonstrated to release an inhibitory peptide tract from the exodomain of the γENaC subunit (Figure 4), either through direct cleavage at high concentrations or via Glycosylphophatidylinositol-anchored prostasin at low concentrations [95,96]. The addition of α2-antiplasmin and aprotinin has been shown to inhibit the ability of nephrotic urine and plasmin to evoke a current [95,96]. Furthermore, the in vitro cleavage of γENaC corresponds to a distinct shift in the migratory pattern of renal tissue γENaC to lower molecular weight isoforms on SDS-PAGE gels in proteinuric conditions [97,98]. The presence of plasminogen immunoreactivity in the urine of preeclampsia (PE) patients with preeclampsia, compared to uncomplicated pregnancies, has prompted the hypothesis that manifest preeclampsia may be linked to plasmin-dependent protease activity in urine [99]. This activity is believed to have the potential to enhance epithelial ENaC activity. The plasmin catalytic activity in PE patients was elevated by about 40-fold compared to normal individuals [99].

Plasmin has been involved in various physiological and pathological processes, including inflammation and tissue remodeling. Its role in ENaC activation suggests that it may contribute to sodium and fluid balance in certain tissues, such as the kidney and lungs.

### 2.5. The Role of Different Cathepsins in ENaC Processing and Activation

Cathepsins are a family of lysosomal proteases involved in the degradation and processing of various proteins [57]. Emerging evidence suggests that specific cathepsins play a role in the processing and activation of ENaC, contributing to the regulation of sodium reabsorption and electrolyte balance [67].

Cathepsins B and S have been identified as key players in ENaC processing and activation. These cathepsins belong to the cysteine protease family and are predominantly localized in endosomes and lysosomes within the cells. Their proteolytic activity can modulate the function and activity of ENaC [61,68,100].

**Figure 5:**
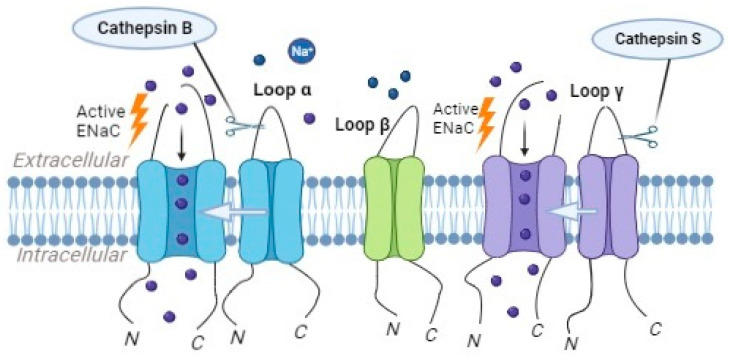
Cathepsin B can activate ENaC, causing the α-subunit to be cleaved, while cathepsin S triggers ENaC activation by cleaving the γ-subunit.

Cathepsin B, in particular, has been implicated in the proteolytic cleavage of the α subunit of ENaC (Figure 5). The cleavage of the α subunit via cathepsin B leads to increased ENaC activity and enhanced sodium transport. Studies have shown that inhibiting cathepsin B’s activity results in reduced ENaC activity and impaired sodium reabsorption [68].

Individuals with nephrotic syndrome frequently exhibit signs of fluid accumulation, such as the development of edema or hypertension. The fundamental disruption in sodium regulation revolves around the improper stimulation of ENaC [67].

In the initial stages of sodium retention in NS, a comprehensive examination revealed the heightened expression of full-length ENaC subunits and the cleaved product of αENaC, accompanied by an increase in ENaC activity, as demonstrated through amiloride application. There was also an upregulation in the expression of Na^+^/K^+^-ATPase in the collecting duct. Urinary proteolytic activity showed an elevation, and mass spectrometry identified several proteases, including cathepsin B, which was determined to be involved in the processing of αENaC. The renal levels of both precursor and active cathepsin B were elevated, with localization observed in glomeruli and intercalated cells. Hypertension was prevented by inhibiting cathepsin B. In conclusion, the identified mechanism involves the cathepsin B-induced processing of αENaC, resulting in heightened channel activity and the development of hypertension [67].

Cystic fibrosis is another disease that shows the combination of ENaC functioning and cathepsin B. In both normal and cystic fibrosis human bronchial epithelial cultures, cathepsin B was found in the apical plasma membrane and the airway surface liquid (ASL). Cathepsin B activity was notably higher in acidic ASL, correlating with an increased presence of ENaC in the plasma membrane and a decrease in the ASL volume. The activation of ENaC, influenced by acid/CTSB, was mitigated by the cell-impermeable, cathepsin B-selective inhibitor CA074, suggesting that inhibiting CTSB might be therapeutically beneficial.

Given that cathepsins primarily reside in lysosomal/endosomal compartments, the site of ENaC processing by cathepsin B poses a challenge. One investigation proposed multiple mechanisms to elucidate the location of ENaC processing by cathepsin B [67]. Cathepsin B undergoes post-translational processing in the rough endoplasmic reticulum, Golgi, endosomal, and lysosomal compartments. Additionally, cathepsin B is secreted into the extracellular space by various tumor epithelial cells. Considering these aspects and in light of the findings that cathepsin B is secreted on the apical side of some epithelial cells, the following several possibilities were considered: cathepsin B cleaving ENaC within the Golgi during post-translational modifications of cathepsin B and ENaC assembly, extracellular cleavage where ENaC is in the apical membrane, or cleavage within endosomes during ENaC recycling [67].

Cathepsin S, another member of the cathepsin family, has been suggested to play a role in the regulation of ENaC activity. Studies have demonstrated that cathepsin S can cleave the γ subunits of ENaC (Figure 5), leading to an increase in the channel’s opening probability and enhanced sodium reabsorption [61]. Mutations in the critical region of γENaC processing by cathepsin S, specifically at valine residues V182 and V193, prevented the proteolytic activation of ENaC by Cathepsin S [61].

The precise regulation and specific roles of cathepsins in ENaC processing and activation are not yet fully understood. However, it is clear that cathepsins, in addition to other proteases, contribute to the proteolytic regulation of ENaC, influencing sodium transport and electrolyte balance.

The activity of cathepsins and their impact on ENaC processing can be modulated by various factors. For example, changes in the pH within the endosomal–lysosomal compartments can influence cathepsin activity and subsequent ENaC activation. Additionally, the presence of endogenous inhibitors, such as cystatins, can regulate cathepsin activity and prevent excessive proteolysis [101].

Further research is needed to elucidate the precise mechanisms by which cathepsins regulate ENaC processing and activation. Understanding these mechanisms could provide valuable insights into the physiological and pathophysiological roles of cathepsins in sodium balance and their potential as therapeutic targets for disorders associated with dysregulated sodium reabsorption.

## 3. Regulation of ENaC Activity by Endogenous Protease Inhibitors

### 3.1. Serine Protease Inhibitors

Serine protease inhibitors, also known as serpins, are a family of endogenous molecules that regulate ENaC activity by inhibiting the serine proteases responsible for ENaC activation. The best-studied serpin in this context is α1-antitrypsin [102]. This protease inhibitor targets the protease prostasin, which processes the γ-subunits of ENaC [103]. By inhibiting prostasin, α1-antitrypsin prevents the cleavage of ENaC subunits, ultimately reducing its activity and sodium reabsorption [104]. The administration of α1-antitrypsin into hypertensive diabetic mice has the potential to restore normal blood pressure in the diabetic kidney. This effect is believed to occur through a mechanism that includes the reduction in renal ENaC and MARCKS protein expression and potentially involves alterations in ceramide metabolism, leading to hexosylceramide within the kidney cells [105].

### 3.2. Kallikrein Inhibitors

Research has shown that kallikrein inhibitor proteins, such as aprotinin, can modulate ENaC activity. Kallikrein inhibitors function by blocking the enzymatic activity of kallikreins, thus indirectly affecting ENaC regulation [76]. Studies have suggested that kallikrein inhibition can reduce ENaC activity and enhance renal sodium excretion, ultimately leading to the lowering of blood pressure [106].

One of the mechanisms through which kallikrein inhibitors influence ENaC activity is by decreasing the proteolytic activation of ENaC subunits. Kallikreins can cleave ENaC subunits, leading to increased channel activity. By inhibiting kallikrein activity, kallikrein inhibitors help maintain a balance in ENaC activation [75].

Kallikrein-like protease activity has been detected on the apical surface and in the apical medium of A6 cells, forming a tight monolayer, as well as in the urinary bladder of toads [107]. The activity of this protease was effectively inhibited by aprotinin at concentrations that also hindered Na^+^ transport in both A6 cells and the toad bladder [108]. Additionally, the introduction of exogenous trypsin and chymotrypsin has been demonstrated to activate INa in epithelia pre-treated with protease inhibitors and induce Na^+^-mediated currents in Xenopus oocytes expressing ENaC [52,109,110,111].

### 3.3. Secretory Leukocyte Protease Inhibitor (SLPI)

SLPI is a multifunctional protein known for its antimicrobial properties [112]. It also plays a role in the regulation of ENaC activity. SLPI inhibits the activity of some serine proteases that activate ENaC, such as prostatic and trypsin [113,114]. By inhibiting these proteases, SLPI limits the cleavage and activation of ENaC, resulting in reduced sodium reabsorption [113].

Moreover, SLPI has been shown to have anti-inflammatory effects [112], and its overexpression has been linked to reduced ENaC activity and a decrease in pro-inflammatory cytokine production [112]. Eliminating SLPI genetically in a murine model of chronic lung disease leads to heightened protease activity and improved mucus clearance [113]. This highlights the potential therapeutic implications of targeting SLPI to manage conditions associated with excess sodium reabsorption and inflammation [112].

### 3.4. Role of Cathepsins Endogenous Inhibitors on the Regulation of ENaC Activity

Recent studies have shown that cathepsins B and S modulate ENaC activity by cleaving ENaC subunits, particularly the γ-subunit. This cleavage can lead to an increase in ENaC activity, promoting sodium absorption across the epithelial membrane. By contrast, when endogenous inhibitors such as cystatins inhibit the activity of cysteine cathepsins, including B and/or S, they prevent the proteolytic cleavage of ENaC subunits, which results in decreased ENaC activity [114].

## 4. Physiological Implications of Proteolytic ENaC Activation

### 4.1. Blood Pressure Regulation

ENaC plays a critical role in the regulation of blood pressure by controlling sodium reabsorption in the kidney [52]. The proteolytic activation of ENaC enhances its activity, leading to increased sodium reabsorption and subsequent volume expansion, which can elevate blood pressure [20,52]. The dysregulation of proteolytic activation can disrupt this fine balance and contribute to the pathogenesis of hypertension [20,52].

### 4.2. Pulmonary Function Regulation

ENaC is critical for maintaining fluid balance and facilitating the clearance of pulmonary edema fluid [26]. The proteolytic activation of ENaC in the airway epithelia by furin-like convertases and tissue kallikreins enhances channel activity and promotes sodium reabsorption, contributing to the reabsorption of fluid from the airway’s surface [101]. This process is crucial for maintaining the integrity of the airway epithelial barrier and preventing the accumulation of excessive fluid in the lung [101]. The dysregulated proteolytic activation of ENaC or the impaired expression of the associated proteases can disrupt fluid clearance mechanisms, leading to pulmonary edema and respiratory dysfunction [20,52].

### 4.3. Intestinal Sodium Absorption

ENaC is responsible for sodium absorption across the epithelial lining [115]. The proteolytic activation of ENaC in the intestinal epithelial cells enables efficient sodium transport and contributes to electrolyte and fluid balance in the gastrointestinal tract [84]. Furin-like convertases and serine proteases have been implicated in the proteolytic processing of ENaC in the intestine [116]. The dysregulation of proteolytic activation can disrupt intestinal sodium absorption, leading to diarrhea or impaired electrolyte absorption [73].

In conclusion, ENaC activation is facilitated by proteases through the cleavage of specific sites in the extracellular domains of the α-, γ-, and δ-subunit, while the β-subunit remains unaffected [53,117,118,119,120,121]. This cleavage process likely leads to the liberation of inhibitory peptides, consequently inducing a conformational change in the channel and activating it. The proteolytic activation of ENaC is critical for its normal activity, and the inhibition of these activations may contribute to certain diseases (Table 1).

## 5. Clinical Relevance and Pharmacological Prospects

Is the regulation of ENaC through proteolysis crucial for maintaining health and managing diseases? Irregularities in ENaC, including its proteolytic processing, are associated with severe human conditions like hypertension, cystic fibrosis, pulmonary edema, pseudohypoaldosteronism type 1, and nephrotic syndrome [27]. The activation of ENaC by proteases is linked to conditions such as renal edema and sodium retention in nephrotic syndrome. Utilizing drugs like amiloride or triamterene to block ENaC might present a more potent alternative to commonly used loop diuretics like furosemide; yet, concerns about potential hyperkalemia accompany this approach. However, there is limited clinical evidence supporting this alternative treatment [122].

In lung cells, for instance, the balance between membrane proteases and protease inhibitors in the solution is believed to regulate ENaC activity, impacting sodium and water absorption in the airways and alveoli [123]. Excessive proteases in the airway liquid contribute to dehydration in cystic fibrosis, while a compromised ENaC cleavage promotes edema formation (Planes 2010). Despite the evidence of ENaC subunit cleavage in human kidneys, the role of proteases in regulating ENaC in the kidneys remains less clear. It is uncertain whether this cleavage represents a specific regulatory mechanism adjusting channel activity for sodium reabsorption.

The accidental contact of proteases with ENaC is a crucial aspect. In acute nephrotic syndrome (NS), serine proteases or their precursor forms, like plasminogen, enter the pre-urine. These active proteases can break down ENaC in the distal nephron, intensifying ENaC’s function, leading to sodium retention, and causing edema—defining characteristics of NS. However, the specific identity of the proteases triggering ENaC activation remains unclear. Moreover, the extent of urinary plasmin’s role in retaining sodium is a topic of ongoing debate in the scientific community [124].

While the clinical and pharmacological implications of ENaC proteolysis are significant for understanding the mechanisms behind certain diseases, further research is crucial to enhance our understanding of the clinical impact of channel proteolysis in this important area.

## 6. Conclusions

The regulation of ENaC activity is crucial for maintaining salt and water balance in the body. Endogenous protease inhibitors like tissue kallikrein, serine protease inhibitors, and SLPI play pivotal roles in this regulation. These inhibitors work by inhibiting the proteases responsible for ENaC activation, ultimately reducing sodium reabsorption.

Understanding the intricate balance between these endogenous protease inhibitors and ENaC is essential for developing targeted therapies for conditions characterized by dysregulated sodium balance, such as hypertension and cystic fibrosis. Further research into the mechanisms of ENaC regulation by these protease inhibitors may reveal new strategies for managing these disorders and maintaining overall physiological homeostasis.

## Figures and Tables

**Table 1:** Proteases involved in ENaC activation and their implications in diseases associated with ENaC.

Protease	Function in ENaC Processing	Examples of Diseases/Conditions
Furin	Cleaves the pro-peptide of ENaC, allowing it to mature and be transported to the cell surface	Hypertension, Liddle syndrome
Cathepsin B	Enhances ENaC activity by cleaving the α-subunit	Asthma, Cystic fibrosis
Cathepsin S	Cleaves the γ-subunit, leading to increased ENaC activity	Cystic fibrosis, Lung diseases
Plasmin	Activates ENaC by cleaving the α-subunit	Hypertension, Edema
Kallikrein	Cleaves the γ-subunit, promoting ENaC activation	Hypertension, Edema
Trypsin	Activates ENaC by cleaving the α-subunit	Cystic fibrosis, Edema
Prostasin	Activates ENaC by cleaving the γ-subunit	Hypertension, Cystic fibrosis
